# Antimicrobial Resistance, Virulence Profiles, and Public Health Significance of *Enterococcus faecalis* Isolated from Clinical Mastitis of Cattle in Bangladesh

**DOI:** 10.1155/2022/8101866

**Published:** 2022-09-27

**Authors:** Md. Abdus Sattar Bag, Mohammad Arif, Sonia Riaz, Md. Shahidur Rahman Khan, Md. Shafiqul Islam, Sadia Afrin Punom, Md. Wohab Ali, Ferdousi Begum, Md. Saiful Islam, Md. Tanvir Rahman, Jayedul Hassan

**Affiliations:** ^1^Department of Microbiology and Hygiene, Faculty of Veterinary Science, Bangladesh Agricultural University, Mymensingh 2202, Bangladesh; ^2^THQ Hospital Karor Lal Esan Layyah, Karor, Karor Lal Esan, Layyah, Pakistan; ^3^Ministry of Fisheries and Livestock, Department of Livestock Services, Kazi Ala-Uddin Road, Dhaka 1100, Bangladesh

## Abstract

This study was designed to identify *Enterococcus faecalis* from clinical mastitis of cattle and determine their antimicrobial resistance and virulence determinants to evaluate their potential public health significance. A total of 105 composite milk samples (80 from cattle with clinical mastitis and 25 from apparently healthy cattle) were analyzed. *E. faecalis* were isolated by culturing on enterococcal selective media and identified by PCR and sequencing. Antimicrobial resistance phenotype was elucidated by the disc diffusion method, and MIC was determined by broth microdilution method according to CLSI guidelines. Detection of antimicrobial resistance and virulence genes was done by PCR. *E. faecalis* were isolated from 11.25% (9/80) of the clinical mastitis and 4% (1/25) of the apparently healthy cattle milk samples. The disc diffusion test revealed 40% isolates as resistant to tetracycline and azithromycin, respectively. Among them, 20% (2/10) of isolates showed resistance to both tetracycline and azithromycin. Tetracycline-resistant isolates showed MIC ranging from ≥64 to >128 *μ*g/ml and carried tetracycline-resistant genes *tetK*, *tetL*, and *tetM* in 25%, 25%, and 50% of the resistant isolates, respectively. On the other hand, all the isolates were sensitive to amoxicillin, ampicillin, bacitracin, chloramphenicol, gentamicin, penicillin, and vancomycin. In addition, the isolates carried at least one of the nine virulence genes screened with *pil* having the highest frequency, followed by *fsrB*, *fsrC*, *ace*, *sprE*, *gelE*, and *agg* genes. Positive correlations were evident between *ace*, *fsrC*, *gelE*, and *sprE* genes that are associated with the attachment and biofilm formation in *E. faecalis. E. faecalis* isolated in this study carried antibiotic resistance and virulence determinants which explain their competence to be potential human pathogens.

## 1. Introduction

Bovine mastitis is one of the most costly production diseases affecting dairy industries globally [1]. In Bangladesh, mastitis was estimated to cause an economic loss of 2.11 million US dollars annually due to reduced milk production and deteriorating milk quality [2]. The disease also has major public health importance because of the possibility of transmission of mastitis-causing pathogen to humans through milk.

Mastitis is caused by a diverse group of organisms that originate from the environment or are transmitted from an infected udder, termed environmental and contagious mastitis, respectively [3]. Control of contagious mastitis has been improved throughout time with well-managed dairy practices, but environmental mastitis remained a major challenge and became the most common and costly form of the disease [3]. *Enterococcus faecalis* is considered as the major environmental mastitis-causing pathogen, and the occurrence of this pathogen was reported as 18% in mastitic cow's milk [4]. Besides, they are potential zoonotic pathogens [5]. *E. faecalis* was reported in animal-originated food including meat, milk, and their products and linked to human diseases such as urinary tract infection [5–10]. Their frequent incidence in food could indicate a zoonotic route for *E. faecalis* transmission to humans preferably through raw milk [11].

Enterococci are well known for having a high level of resistance against a wide variety of antimicrobial substances, developed by both intrinsic and acquired mechanisms. They are intrinsically resistant to virtually all cephalosporins, aminoglycosides, clindamycin, and trimethoprim-sulfamethoxazole [12]. *E. faecalis* are intrinsically susceptible to carbapenems, vancomycin, tetracycline, and fluoroquinolones; moreover, acquired resistance to the antibiotics through transposons or plasmids has been reported [12]. Due to their evolving resistance, mastitis caused by *E. faecalis* might be difficult to treat with most commercial antimicrobials.

In addition to antimicrobial resistance, virulence factors contribute to the pathogenesis of *E. faecalis*. Many researchers have reported several virulence factors in *E. faecalis* that might be involved in the severity of diseases in humans and animals [13]. Studies have reported the presence of virulence factors that facilitate adherence and colonization (*agg, ace*) and cytolysis and dissemination of *E. faecalis* into the host (*cyl, gelE,* and *sprE*) [14]. Furthermore, *E. faecalis* has biofilm-forming machinery (*pili, gelE,* and fsr quorum-sensing systems) that allows the bacterium to adhere to biotic and abiotic surfaces and confers additional antimicrobial resistance [14]. The multifaceted virulence determinants enable this opportunistic pathogen to cause infections in the urinary tract, skin, soft tissue, abdomen, pelvis, central nervous systems, etc. in humans [15]. As enterococci can easily spread antimicrobial resistance or virulence genes to other bacterial species via horizontal transfer, the presence of enterococci in milk can enhance the emergence of MDR, transfer pathogenic *E. faecalis* to humans, and eventually affect the choice of drug [16].

To the best of our knowledge, no data yet exist from Bangladesh on the evaluation of antimicrobial resistance and virulence patterns in *E. faecalis* isolated from bovine clinical mastitis. Therefore, we aimed to conduct the present study to determine the antimicrobial resistance and virulence determinants in *E. faecalis* isolated from clinical mastitis of cattle having potential public health significance.

## 2. Materials and Methods

### 2.1. Sampling Site and Sampling

The present study was conducted in Dhaka (23.8105°N, 90.3372°E), Mymensingh (24.7539°N, 90.4073°E), and Sirajganj (24.3141°N, 89.5700°E) districts of Bangladesh between January 2019 and June 2021. A total of 105 milk samples were collected comprising 80 from cattle with clinical mastitis and 25 apparently healthy cattle. Cow with clinical mastitis was identified by the residential farm veterinarian based on gross changes in the udder (redness, swelling, and sensitive udder) and/or milk (flakes and/or clots). For the identification of apparently healthy cattle, the California Mastitis Test was used [17]. Cow's milk showing a negative CMT score, i.e., no visible precipitate on paddle movement, was considered apparently healthy. The milk samples were collected from the major dairy farms having a history of persistent mastitis. During a single visit to each farm, 10 ml of composite milk sample was aseptically collected by the residential veterinarian directly from the udder of a cow and transported to the laboratory in an icebox for microbiological analysis.

### 2.2. Isolation and Identification of *E. faecalis*

Enrichment of milk samples was performed in Luria Bertani (LB) broth as described earlier [18]. 100 *μ*l of the enriched sample was spread onto modified Edwards medium (MEM) (Himedia, India) and incubated aerobically overnight at 37°C. Black color colonies characteristic of *Enterococcus* spp. obtained on MEM were screened for bacterial morphology by Gram's staining. At least three colonies showing characteristics of *Enterococcus* spp. were purified by subsequent streaking onto MEM. Crude genomic DNA was extracted from the purified colonies by boiling method and subjected to PCR targeting *ddl* gene [19] for the identification of *E. faecalis* using the primers provided in Supplementary Table S1. PCR reaction was adjusted to 20 *μ*l volume with 10 *μ*l 2X GoTaq® G2 Green Master Mix (Promega, USA), 10 pmol of each primer (Supplementary Table S1), and 2 *μ*l of DNA templates. PCR was conducted in an ASTEC 482 thermal cycler (Japan) with an initial denaturation at 95°C for 5 min followed by 30 cycles of denaturation at 95°C for 30 sec, annealing at 54°C for 30 sec, extension at 72°C for 1 min, and a final extension step at 72°C for 5 min. Representative isolates which were positive for the *ddl* gene were further confirmed by sequencing of 16S rRNA using the primers 8F and 1492R [20] (Supplementary Table S1).

### 2.3. Antimicrobial Susceptibility Testing

Disc diffusion method [21] was performed to determine the susceptibility of *E. faecalis* isolates to antimicrobials commonly used to treat animal and human diseases including mastitis in Bangladesh. The results (resistant and susceptible) were interpreted following the guidelines of the Clinical and Laboratory Standards Institute [22]. A total of nine (9) different antimicrobials (Oxoid, UK) were employed such as *β*-lactams (amoxicillin 10 *μ*g (AMX), ampicillin 10 *μ*g (AMP), and penicillin G 10 *μ*g (P)), aminoglycosides (gentamicin 30 *μ*g (GEN)), glycopeptides (vancomycin 30 *μ*g (VA)), macrolides (azithromycin 15 *μ*g (AZM)), phenicols (chloramphenicol 30 *μ*g (C)), polypeptides (bacitracin 10 units (B)), and tetracyclines (tetracycline 30 *μ*g (TE)). *E. coli* strain ATCC25922 was used as the control bacterial strain, and kanamycin (30 *μ*g) was used as the control antibiotic in each experiment. Each experiment was performed three times to confirm the reproducibility of the results. Isolates resistant to three or more antimicrobial classes were considered multidrug resistant (MDR) [23].

### 2.4. Minimum Inhibitory Concentration

All the isolates were subjected to broth microdilution, to determine the minimum inhibitory concentration (MIC) against gentamicin (FUJIFILM Wako Pure Chemical Corporation, Tokyo, Japan) and tetracycline (Nacalai Tesque Inc., Kyoto, Japan) following CLSI guidelines [22]. Test plates consisted of 2-fold dilutions of gentamicin and tetracycline ranging from 2 to 512 *μ*g/ml and from 0.5 to 128 *μ*g/ml, respectively. Plates were interpreted according to CLSI guidelines, and MIC breakpoint of each sample against their respective antibiotic was recorded where growth was significantly reduced, ignoring tiny buttons or light or faint turbidity [22]. Each test was performed three times to examine its reproducibility. *E. coli* ATCC 25922 was included in each trial as the quality control strain. Isolates exhibiting MIC > 500 *μ*g/ml gentamicin are considered as high-level gentamicin resistance (HLGR), otherwise considered as wild or low-level resistance to gentamicin [22].

### 2.5. Detection of Antimicrobial Resistance Genes

*E. faecalis* isolates showing antimicrobial resistance were screened for the presence of antimicrobial-resistant genes by PCR. Genes conferring resistance to tetracycline (*tetA*, *tetB*, *tetC*, *tetD*, *tetE*, *tetG*, *tetK*, *tetL*, and *tetM*) were screened using the primers enlisted in Supplementary Table S1 and protocols described earlier [24–26]. In addition, genes conferring resistance to gentamicin *(aac*(6′)−*Ie* − *aph(2^″^)* − *Ia*, *aph*(2^″^) − *Ib*, *aph*(2^″^) − *Ic*, *aph*(2^″^) −*Id*, *aph*(3^″^) −*IIIa*, *aacC2* and *aacC4*) and vancomycin (*vanA*, *vanB*) were screened considering current trends of gentamicin and vancomycin resistance in *Enterococcus* spp. [27–29]. Briefly, the PCR reaction mixture was adjusted to 20 *μ*l with 20 *μ*l 2x GoTaq® G2 Green Master Mix (Promega, USA), 20 pmol of each primer (Supplementary Table S1), and 2 *μ*l of DNA templates. PCR was conducted in an ASTEC 482 thermal cycler (Japan) with an initial denaturation at 95°C for 5 min followed by 30 cycles of denaturation at 95°C for 30 sec, annealing for 1 min at different temperatures according to the primers used (Supplementary Table S1), extension at 72°C for 1 min, and a final extension step at 72°C for 7 min. In all PCR amplifications, *E. coli* strain ATCC25922 was used as the negative control.

### 2.6. Detection of Virulence Genes

*E. faecalis* isolated in this study were PCR screened for the presence of virulence genes previously described in human clinical isolates [14]. A total of nine virulence genes were screened including *ace*, *agg*, *cyl*, *fsrA*, *fsrB*, *fsrC*, *gelE*, *pil*, and *sprE*. Primers used for this study are enlisted in Supplementary Table S1. PCR reaction mixture and PCR conditions were followed as per Section 2.2 except for the annealing temperature, which was varied depending on the primers used (Supplementary Table S1).

### 2.7. Sequencing and Analysis

16S rRNA gene of the representative *E. faecalis* isolates was sequenced using 8F and 1492R primers (Supplementary Table S1) on an Applied Biosystems 3500 series genetic analyzer (Thermo Fisher Scientific, USA) using Sanger's dideoxy sequencing technique [18]. Confirmation of detection of the *E. faecalis* sequences was done by BLAST search (http://blast.ncbi.nlm.nih.gov/Blast.cgi) and submitted to GenBank.

### 2.8. Statistical Analysis

Data obtained from this study were incorporated into Excel 2013 (Microsoft Office 2013, Microsoft, Los Angeles, CA, USA) and then transferred to statistical tools for further analysis.

#### 2.8.1. Descriptive Analysis

By the Statistical Package for the Social Sciences (SPSS) software (IBM SPSS version 25.0, USA), a Fisher's exact test was conducted to find the possible variations between the occurrences of *E. faecalis*. A *p* value less than 0.05 was considered statistically significant in all tests.

#### 2.8.2. Bivariate Analysis

Pearson correlation coefficient was enumerated by a bivariate analysis to assess the correlations between antimicrobials resistant to isolated *E. faecalis*, to evaluate the associations between resistance genes of *E. faecalis* isolates, and finally to determine the interrelation between different virulence genes of *E. faecalis* isolates. A statistically significant *p* value was less than 0.05. The bivariate analysis was performed in SPSS software (version 25).

## 3. Results

### 3.1. Occurrence of *E. faecalis*

**Figure 1:**
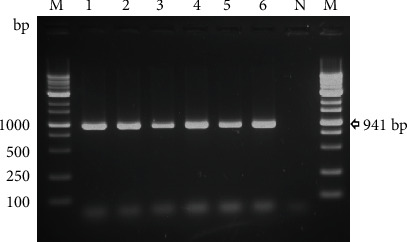
Representative photograph of PCR targeting *E. faecalis* specific *ddl* gene. Lanes: 1-6: suspected *E. faecalis* colonies; N: *E. coli* strain ATCC25922 (negative control); M: 1 kb DNA ladder, Promega. Electrophoresis was performed with 1.5% LE agarose (Promega) at 100 volts for 25 minutes in 1x TAE buffer.

In the present study, 9.52% (10/105) of milk samples were positive for *E. faecalis* following culture and PCR targeting the *ddl* gene (Figure 1, Table 1). Representatives of the isolates were further confirmed by sequencing, BLASTn homology, and phylogenetic analysis of the 16S rRNA (Supplementary Figure S1). The sequences were submitted to GenBank (GenBank accession numbers OK187180, OK187181, OK187182, OK187183, OK187184, and OK187185). The occurrence of *E. faecalis* in clinical mastitis samples (11.25%, 9/80) was higher than milk samples originating from apparently healthy cattle (4%, 1/25); however, the difference was not statistically significant (*p* = 0.281) (Table 1).

### 3.2. Antimicrobial Susceptibility Patterns of the Isolated *E. faecalis*

#### 3.2.1. Antimicrobial Susceptible Phenotypes

In the antibiogram, *E. faecalis* isolates were found sensitive to the entire nine antibiotics tested except tetracycline and azithromycin. Forty percent (40%) of the isolates were found sensitive to tetracycline and azithromycin, respectively (Table 2). None of the isolates was multidrug resistant. In MIC test, the MIC of the isolates to gentamicin was ≥8-16 *μ*g/ml confirming their sensitivity to this antibiotic. On the other hand, tetracycline-resistant isolates revealed a MIC of ≥64-128 *μ*g/ml (Table 2). Bivariate analysis was performed to elucidate any correlation in phenotypic resistance; however, no positive significant correlation was observed between tetracycline and azithromycin resistance (Table 3).

#### 3.2.2. Antimicrobial Resistance Genotypes

By PCR, 60% of the *E. faecalis* isolates harbored at least one out of 18 antimicrobial resistance genes examined. Tetracycline-resistant isolates carried *tetK*, *tetL*, and *tetM* as 25% (1/4), 25% (1/4), and 50% (2/4), respectively (Table 2). Although all the isolates were sensitive to gentamicin and vancomycin, aminoglycoside-resistant genes *aph*(3^″^) − *IIIa*, *aacC2*, and *aacC4* were detected in 12.5% (1/8), 12.5% (1/8), and 37.5% (3/8) of the isolates, respectively; and one (1) of the isolates carried *vanB* gene. Besides, none of the antibiotic-resistant gene was detected in four (4) isolates examined. In bivariate analysis, high positive significant correlations were observed in between *aac2* and *tetM* (*p* = 0.035), and *aph*(3^″^) − *IIIa* and *tetL gene* (*p* = 0.001) (Table 4).

### 3.3. Distribution of the Virulence Genes in the Isolated *E. faecalis*

All the *E. faecalis* isolates were found positive for at least one virulence gene, where 80% (8/10) of the isolates harbored four or more virulence genes. Occurrence of *pil* gene (100%) was higher than virulence genes *fsrB* (80%), *fsrC* (60%), *ace* (60%), *sprE* (30%), *gelE* (20%), and *agg* (10%) (Table 2). However, no isolates carried virulence genes *fsrA* and *cyl*. Based on bivariate analysis, high and moderate positive significant correlations were revealed in between *ace* and *fsrC* (*p* = 0.001), and *gelE* and *sprE* (*p* = 0.01) virulence genes of *E. faecalis*. On the other hand, a negative moderate significant correlation was observed between *agg* and *fsrB* (*p* = 0.035) virulence genes of *E. faecalis* (Table 5).

## 4. Discussion

Antimicrobial resistance is a global public health concern. Indiscriminate or irrational use of medically important antimicrobials in animal production is claimed as a major driver of antimicrobial resistance transfer to humans [30]. In animal production, dairy farms are the largest user of medically important antimicrobial where mastitis comprises the single most common cause. Bovine mastitis is one of the challenging veterinary infections to control, and enterococci are important causative pathogens for mastitis. Over time, enterococci represent one of the most significant pathogens to cause infections, especially in humans, by acquiring antimicrobial resistance and virulence determinants [31]. *E. faecalis* are the most common enterococci species in clinical mastitis; however, there is no study yet in Bangladesh in detecting antimicrobial resistance and virulence factors of this organism from clinical bovine mastitis. From this perspective, we undertook the present study to identify *E. faecalis* with their antimicrobial resistance, corresponding resistance genes, and virulence factors from cattle showing mastitis.

In this study, the occurrence rate of *E. faecalis* in milk samples of cattle having mastitis was 11.25% which is higher than previously reported as 0.2% in Germany and the United States [32, 33]. However, a higher prevalence of *E. faecalis* in clinical mastitis was reported in Belgium (20%) and South Korea (86.5%) [34, 35]. The observed differences in occurrence might be attributed to the geographical location and sample sizes. However, the presence of *E. faecalis* in milk samples represents a threat to human health as they can be transmitted to humans via the consumption of contaminated milk or milk products [11].

Tetracycline is one of the most commonly used antimicrobials in animal production for disease control and growth promotion. Widespread uses of this antimicrobial lead to the emergence of tetracycline-resistant bacteria [36]. In addition to tetracycline, use of azithromycin is increasingly reported in animal production in Bangladesh (personal communication). Besides, tetracycline and azithromycin are among the most frequently prescribed antibiotics for human diseases in Bangladesh [37]. Thus, the occurrence of tetracycline and azithromycin resistance in *E. faecalis* is quite alarming since it could lead to treatment failure and potential life-threatening diseases in humans if proper antimicrobials are not selected. Interestingly, the isolates described in this study were sensitive to seven (7) other antibiotics tested including penicillin and gentamicin. Gentamicin is the most widely used aminoglycosides against enterococci; however, due to the emergence of HLGR, gentamicin monotherapy becomes ineffective in such cases. In case of low-level resistance, gentamicin or other aminoglycosides are suggested in combination therapy with cell wall inhibitors like penicillin and glycopeptides against *Enterococcus* [22, 38]. Based on the findings of this study, combination therapy could be suggested in controlling mastitis caused by *E. faecalis* in the study areas. However, antibiotic sensitivity testing is warranted before prescribing any antibiotics to control mastitis as a diverse group of microorganisms are involved in this disease pathogenesis.

Tetracycline resistance is conferred by diverse tetracycline-resistant genes located on horizontally transferable elements. At least thirty different tetracycline-resistant genes have been described so far [36, 39]. In this study, we have screened the *E. faecalis* isolates for nine (9) different tetracycline-resistant genes where the isolates carried either of the three tetracycline-resistant genes *tetK* or *tetL* or *tetM*, indicating the diversity of the tetracycline-resistant genes in the study area. Detection of *tetK*, *tetL*, and *tetM* genes in *E. faecalis* isolated from subclinical or clinical bovine mastitis has been reported earlier [40, 41]. However, ascertaining the real diversity of tetracycline-resistant genes in *E. faecalis* isolated from mastitis in Bangladesh needs further investigation with more samples and isolates.

Antibiotic resistance is conferred by a diverse mechanism including the presence of respective genes or bacterial metabolism [42, 43]. However, the presence of an antibiotic-resistant gene does not always mean that it would confer resistance to the respective antibiotic due to alteration through mutation or other genetic mechanisms [44]. In this study, we have detected plasmid-encoded aminoglycoside N(3)-acetyltransferases II and IV encoding genes (*aacC2* and *aacC4*) in some of the *E. faecalis*. These genes are known to confer resistance to gentamicin [45, 46]. However, the isolates did not show gentamicin resistance nor their presence was previously described in other studies. Thus, further studies are required to ascertain the presence and role of these genes in *E. faecalis* pathogenicity. In addition, one of the *E. faecalis* isolates carried *vanB* gene despite phenotypic vancomycin sensitivity indicating that the gene is dormant or nonfunctional in this strain. The presence of *aacC2*, *aacC4*, and *vanB* genes might not be associated with antibiotic resistance in *E. faecalis* isolated in this study but poses a threat of transferring these resistance determinants to other enterobacteria having clinical significance in human infections.

Pathogenesis of *E. faecalis* is dependent on its establishment, adherence, invasiveness, and ability to overcome the host defense system and biofilm formation—an important attribute of the bacteria that facilitates its persistence in adverse environmental conditions [47]. In this study, all the *E. faecalis* isolates were positive for virulence gene *pili* and six for *ace* genes. The *pili* and *ace* are two important virulence factors whose products are associated with adhesion and colonization in the host [14]. In addition, our present study found that *E. faecalis* isolates also carried virulence genes *fsrB*, *fsrC*, *sprE*, and *gelE*, whose products are linked to biofilm formation and its strength in *E. faecalis* [14]. Furthermore, a moderate to strong association between the *ace* and *fsr* genes, as well as the *sprE* and *gelE* genes, indicates that the isolated *E. faecalis* are strong biofilm former, as evidenced by previous studies [14]. It is clear from our findings that the *E. faecalis* isolates have the characteristics of a potential human pathogen. In addition, these virulence properties might be linked to *E. faecalis* persistence in the udder environment. However, to ascertain these possibilities, phenotypic expression of the virulence properties is suggested, which could not be performed due to a lack of our laboratory facilities and funding.

## 5. Conclusion

For the first time in Bangladesh, in this study, we have detected antimicrobial resistance and virulence determinants of *E. faecalis* from bovine clinical mastitis. The occurrence of these resistant isolates in milk samples from mastitis as well as apparently healthy cattle is of public health concern. *E. faecalis* isolated in this study might be strong biofilm formers. Their biofilm-associated virulence determinants and antimicrobial resistance could be the reason for their persistence in the udder environment and resistance to antimicrobial therapy. Their virulence properties and resistance to medically important antimicrobials indicate their potential to induce human or animal diseases that might be difficult to treat if proper antimicrobials are not selected. Thus, an antibiotic sensitivity test is suggested before prescribing any antimicrobials for mastitis.

## Figures and Tables

**Table 1:** Occurrence of *E. faecalis* in milk samples.

Health status	No. of samples (*n*)	No. (%) of positive *E. faecalis*	*p* value^∗^
Clinical mastitis	80	9 (11.25)	0.281
Apparently healthy	25	1 (4)	

*n* = number of samples to be tested ^∗^. A *p* value less than 0.05 (*p* < 0.05) was regarded as significant.

**Table 2:** Antimicrobial resistance and virulence properties of the *E. faecalis* isolated in this study.

Isolate ID	Sources	Antibiotic resistance				Virulent genes
		Phenotypes	Genotypes	MIC (*μ*g/ml)		
				GEN	TE	
2006	MCCM			≥8.0	<1.0	*ace, fsrB, fsrC, pil*
2008	MCCM			<2.0	<1.0	*ace, fsrB, fsrC, pil*
2020	MCCM	AZM, TE	*tetK*	≥16	≥64	*ace, fsrB, fsrC, pil*
2024	MCCM			≥16	<1.0	*ace, fsrB, fsrC, pil*
2029	MCCM			<2.0	<1.0	*ace, fsrB, fsrC, pil*
2032	MCCM	AZM, TE	*tetL, aph*(3^″^) − *IIIa*	≥16	≥128	*ace, fsrB, fsrC, sprE, pil*
2073	MCCM	TE	*aacC4, tetM,*	≥8.0	≥64	*agg, pil*
2081	MCCM	TE	*aacC2, tetM*	≥8.0	≥64	*pil*
2091	MCCM	AZM	*aacC4, vanB*	≥8.0	<1.0	*fsrB, gelE, sprE, pil*
20105	MAHC	AZM	*aacC4*	≥16	<1.0	*fsrB, gelE, sprE, pil*

MCCM: milk of cattle with clinical mastitis; MAHC: milk of apparently healthy cattle; AZM: azithromycin; GEN: gentamicin; TE: tetracycline.

**Table 3:** Correlation between the phenotypic resistances of different antimicrobials against *E. faecalis*.

		TE	AZM
TE	Pearson correlation	1	
	Sig. (2-tailed)		
AZM	Pearson correlation	0.167	1
	Sig. (2-tailed)	0.645	

TE: tetracycline; AZM: azithromycin.

**Table 4:** Correlation between antimicrobial resistance genes detected in the *E. faecalis* recovered in this study.

		*aacC2*	*aacC4*	*tetK*	*tetL*	*tetM*	*aph(3*′*)-IIIa*	*vanB*
*aacC2*	Pearson correlation	1						
	Sig. (2-tailed)	—						
*aacC4*	Pearson correlation	-0.218	1					
	Sig. (2-tailed)	0.545	—					
*tetK*	Pearson correlation	-0.111	-0.218	1				
	Sig. (2-tailed)	0.76	0.545	—				
*tetL*	Pearson correlation	-0.111	-0.218	-0.111	1			
	Sig. (2-tailed)	0.76	0.545	0.76	—			
*tetM*	Pearson correlation	**0.667** ^∗^	0.218	-0.167	-0.167	1		
	Sig. (2-tailed)	**0.035**	0.545	0.645	0.645	—		
*aph(3*″*)-IIIa*	Pearson correlation	-0.111	-0.218	-0.111	**1.000** ^∗∗^	-0.167	1	
	Sig. (2-tailed)	0.76	0.545	0.76	**0.001**	0.645	—	
*vanB*	Pearson correlation	-0.111	0.509	-0.111	-0.111	-0.167	-0.111	1
	Sig. (2-tailed)	0.76	0.133	0.76	0.76	0.645	0.76	—

A *p* value less than 0.05 was deemed statistically significant ^∗^. Correlation is significant at the 0.05 level (2-tailed) ^∗∗^. Correlation is significant at the 0.01 level (2-tailed).

**Table 5:** Correlation between the occurrences of virulence genes in the *E. faecalis* isolated in this study.

		*agg*	*fsrB*	*fsrC*	*gelE*	*sprE*	*ace*	*pil*
*agg*	Pearson correlation	1						
	Sig. (2-tailed)	—						
*fsrB*	Pearson correlation	**-0.667** ^∗^	1					
	Sig. (2-tailed)	**0.035**	—					
*fsrC*	Pearson correlation	-0.408	0.612	1				
	Sig. (2-tailed)	0.242	0.06	—				
*gelE*	Pearson correlation	-0.167	0.25	-0.612	1			
	Sig. (2-tailed)	0.645	0.486	0.06	—			
*sprE*	Pearson correlation	-0.218	0.327	-0.356	**0.764** ^∗^	1		
	Sig. (2-tailed)	0.545	0.356	0.312	**0.01**	—		
*ace*	Pearson correlation	-0.408	0.612	**1.000** ^∗∗^	-0.612	-0.356	1	
	Sig. (2-tailed)	0.242	0.06	**0.001**	0.06	0.312	—	
*pil*	Pearson correlation	.a	.a	.a	.a	.a	.a	.a
	Sig. (2-tailed)	—	—	—	—	—	—	—

A *p* value less than 0.05 was deemed statistically significant ^∗^. Correlation is significant at the 0.05 level (2-tailed) ^∗∗^. Correlation is significant at the 0.01 level (2-tailed).

## Data Availability

The data will be available from the corresponding author on request.

## Supplementary Materials

Supplementary Materials Supplementary Table S1: list of primers used for the detection of antimicrobial resistance and virulent genes in the *E. faecalis* isolated from clinical mastitis. Supplementary Figure S1: phylogenetic relationship of *E. faecalis* isolated in this study. The relationship was inferred with the partial 16S rRNA gene sequences of *E. faecalis* (marked with red rectangles) obtained in this study using the Neighbor-Joining method (Saitu and Nei, 1987). The optimal tree is shown. The percentage of replicate trees in which the associated taxa clustered together in the bootstrap test (1000 replicates) is shown next to the branches (Felsenstein, 1985). The tree is drawn to scale, with branch lengths in the same units as those of the evolutionary distances used to infer the phylogenetic tree. The evolutionary distances were computed using the p-distance method (Nei and Kumar, 2000) and are in the units of the number of base differences per site. This analysis involved 15 *E. faecalis* and 1 *E. coli* sequence as an out-group. All ambiguous positions were removed for each sequence pair (pairwise deletion option). There were a total of 1452 positions in the final dataset. Evolutionary analyses were conducted in MEGA X (Kumar et al., 2018).

## References

[1] Gomes F., Henriques M. (2016). Control of bovine mastitis: old and recent therapeutic approaches. *Current Microbiology.*.

[2] Kader M. A., Samad M. A., Saha S. (2003). Influence of host level factors on prevalence and economics of subclinical mastitis in dairy milch cows in Bangladesh. *Indian Journal of Dairy Science*.

[3] Klaas I. C., Zadoks R. N. (2018). An update on environmental mastitis: challenging perceptions. *Transboundary and Emerging Diseases*.

[4] Różańska H., Lewtak-Piłat A., Kubajka M., Weiner M. (2019). Occurrence of enterococci in mastitic cow’s milk and their antimicrobial resistance. *Journal of Veterinary Research*.

[5] Abat C., Huart M., Garcia V., Dubourg G., Raoult D. (2016). _Enterococcus faecalis_ urinary-tract infections: do they have a zoonotic origin?. *Journal of Infection*.

[6] Franz C. M., Holzapfel W. H., Stiles M. E. (1999). Enterococci at the crossroads of food safety?. *International Journal of Food Microbiology*.

[7] Francesca N., Sannino C., Moschetti G., Settanni L. (2013). Microbial characterisation of fermented meat products from the Sicilian swine breed “Suino Nero Dei Nebrodi”. *Annals of Microbiology*.

[8] Hasan K. A., Ali S. A., Rehman M., Bin-Asif H., Zahid S. (2018). The unravelledEnterococcus faecaliszoonotic superbugs: emerging multiple resistant and virulent lineages isolated from poultry environment. *Zoonoses and Public Health*.

[9] Olsen R. H., Schønheyder H. C., Christensen H., Bisgaard M. (2012). Enterococcus faecalis of human and poultry origin share virulence genes supporting the zoonotic potential of E. faecalis. *Zoonoses and Public Health*.

[10] Devriese L. A., Pot B., Van Damme L., Kersters K., Haesebrouck F. (1995). Identification of _Enterococcus_ species isolated from foods of animal origin. *International Journal of Food Microbiology*.

[11] Gelsomino R., Vancanneyt M., Cogan T. M., Condon S., Swings J. (2002). Source of enterococci in a farmhouse raw-milk cheese. *Applied and Environmental Microbiology*.

[12] García-Solache M., Rice L. B. (2019). The enterococcus: a model of adaptability to its environment. *Clinical Microbiology Reviews*.

[13] Chajęcka-Wierzchowska W., Zadernowska A., Łaniewska-Trokenheim L. (2017). Virulence factors of _Enterococcus_ spp. presented in food. *LWT-Food Science and Technology*.

[14] Hashem Y. A., Abdelrahman K. A., Aziz R. K. (2021). Phenotype–genotype correlations and distribution of key virulence factors in Enterococcus faecalis isolated from patients with urinary tract infections. *Infection and Drug Resistance*.

[15] O’Driscoll T., Crank C. W. (2015). Vancomycin-resistant enterococcal infections: epidemiology, clinical manifestations, and optimal management. *Infection and Drug Resistance*.

[16] Gao X., Fan C., Zhang Z. (2019). Enterococcal isolates from bovine subclinical and clinical mastitis: antimicrobial resistance and integron-gene cassette distribution. *Microbial Pathogenesis*.

[17] Peek S. F., Divers T. J. (2017). *Rebhun’s Diseases of Dairy Cattle 3^rd^ Edn*.

[18] Bag M. A. S., Khan M. S. R., Sami M. D. H. (2021). Virulence determinants and antimicrobial resistance of _E. coli_ isolated from bovine clinical mastitis in some selected dairy farms of Bangladesh. *Saudi Journal of Biological Sciences*.

[19] Dutka-Malen S., Evers S., Courvalin P. (1995). Detection of glycopeptide resistance genotypes and identification to the species level of clinically relevant enterococci by PCR. *Journal of Clinical Microbiology*.

[20] Hahne J., Kloster T., Rathmann S., Weber M., Lipski A. (2018). Isolation and characterization of Corynebacterium spp. from bulk tank raw cow's milk of different dairy farms in Germany,. *PLoS One*.

[21] Bauer A., Kirby W., Sherris J., Turch M. (1966). Antibiotic susceptibility testing by a standardized single disk method. *American Journal of Clinical Pathology*.

[22] CLSI, Wayne P. A. (2020). Performance Standards for Antimicrobial Susceptibility Testing. 30^th^ ed. CLSI supplement M100. *Clinical and Laboratory Standards Institute*.

[23] Magiorakos A. P., Srinivasan A., Carey R. B. (2012). Multidrug-resistant, extensively drug-resistant and pandrug-resistant bacteria: an international expert proposal for interim standard definitions for acquired resistance. *Clinical Microbiology and Infection*.

[24] Aarestrup F. M. (2000). Occurrence, selection and spread of resistance to antimicrobial agents used for growth promotion for food animals in Denmark. *Acta Pathologica, Microbiologica, et Immunologica Scandinavica. Supplementum*.

[25] Trzcinski K., Cooper B. S., Hryniewicz W., Dowson C. G. (2000). Expression of resistance to tetracyclines in strains of methicillin-resistant Staphylococcus aureus. *Journal of Antimicrobial Chemotherapy*.

[26] Chen S., Zhao S., White D. G. (2004). Characterization of multiple-antimicrobial-resistantSalmonellaserovars isolated from retail meats. *Applied and Environmental Microbiology*.

[27] Clark N. C., Cooksey R. C., Hill B. C., Swenson J. M., Tenover F. C. (1993). Characterization of glycopeptide-resistant enterococci from U.S. hospitals. *Antimicrobial Agents and Chemotherapy*.

[28] Saha B., Singh A. K., Ghosh A., Bal M. (2008). Identification and characterization of a vancomycin-resistant Staphylococcus aureus isolated from Kolkata (South Asia). *Journal of Medical Microbiology*.

[29] Vakulenko S. B., Donabedian S. M., Voskresenskiy A. M., Zervos M. J., Lerner S. A., Chow J. W. (2003). Multiplex PCR for detection of aminoglycoside resistance genes in enterococci. *Antimicrobial Agents and Chemotherapy*.

[30] Ma F., Xu S., Tang Z., Li Z., Zhang L. (2021). Use of antimicrobials in food animals and impact of transmission of antimicrobial resistance on humans. *Biosafety and Health*.

[31] Kristich C. J., Rice L. B., Arias C. A. (2014). Enterococcal infection—treatment and antibiotic resistance,In, Enterococci: From Commensals to Leading Causes of Drug Resistant Infection [Internet]. *Boston: Massachusetts Eye and Ear Infirmary*.

[32] Tenhagen B. A., Köster G., Wallmann J., Heuwieser W. (2006). Prevalence of mastitis pathogens and their resistance against antimicrobial agents in dairy cows in Brandenburg, Germany. *Journal of Dairy Science*.

[33] Petersson-Wolfe C. S., Adams S., Wolf S. L., Hogan J. S. (2008). Genomic typing of enterococci isolated from bovine mammary glands and environmental sources^1^. *Journal of Dairy Science*.

[34] Devriese L. A., Hommez J., Laevens H., Pot B., Vandamme P., Haesebrouck F. (1999). Identification of aesculin-hydrolyzing streptococci, lactococci, aerococci and enterococci from subclinical intramammary infections in dairy cows. *Veterinary Microbiology*.

[35] Kim H. J., Youn H. Y., Kang H. J. (2022). Prevalence and virulence characteristics of Enterococcus faecalis and Enterococcus faecium in bovine mastitis milk compared to bovine normal raw milk in South Korea. *Animals (Basel)*.

[36] Michalova E., Novotna P., Schlegelova J. (2004). Tetracyclines in veterinary medicine and bacterial resistance to them. *Veterinární Medicína*.

[37] Akhtar Z., Mah-E-Muneer S., Rashid M. M. (2021). Antibiotics use and its knowledge in the community: a mobile phone survey during the COVID-19 pandemic in Bangladesh. *Antibiotics*.

[38] El-Ghazawy I. F., Okasha H. A., Mazloum S. M. (2016). A study of high level aminoglycoside resistant enterococc. *African Journal of Microbiology Research*.

[39] Yoon S., Lee Y. J. (2021). Molecular characteristics of Enterococcus faecalis and Enterococcus faecium from bulk tank milk in Korea. *Animals*.

[40] Goksel E., Ugur P., Suheyla T., Nese U., Mehmet O., Osman K. (2016). Distribution of antibiotic resistance genes in Enterococcus spp. isolated from mastitis bovine milk. *Acta Veterinaria-Beogard*.

[41] Yang F., Zhang S., Shang X. (2019). Short communication:_ antimicrobial resistance and virulence genes of _Enterococcus faecalis_ isolated from subclinical bovine mastitis cases in China. *Journal of Dairy Science*.

[42] Bennett P. M. (2008). Plasmid encoded antibiotic resistance: acquisition and transfer of antibiotic resistance genes in bacteria. *British Journal of Pharmacology*.

[43] Corona F., Martinez J. L. (2013). Phenotypic resistance to antibiotics. *Antibiotics (Basel)*.

[44] Amer M. M., Mekky M. H., Amer A. M., Fedawy H. S. (2018). Antimicrobial resistance genes in pathogenic Escherichia coli isolated from diseased broiler chickens in Egypt and their relationship with the phenotypic resistance characteristics. *Veterinary World*.

[45] Vliegenthart J. S., Ketelaar-van Gaalen P. A., Van De Klundert J. A. (1989). Nucleotide sequence of the aacC2 gene, a gentamicin resistance determinant involved in a hospital epidemic of multiply resistant members of the family Enterobacteriaceae. *Antimicrobial Agents and Chemotherapy*.

[46] Bräu B., Pilz U., Piepersberg W. (1984). Genes for gentamicin-(3)-N-acetyltransferases III and IV:. *Molecular and General Genetics*.

[47] Zheng J. X., Wu Y., Lin Z. W. (2017). Characteristics of and virulence factors associated with biofilm formation in clinical Enterococcus faecalis isolates in China. *Frontiers in Microbiology*.

