# Supplementary material for: Antimicrobial Resistance, Virulence Profiles, and Public Health Significance of Enterococcus faecalis Isolated from Clinical Mastitis of Cattle in Bangladesh
Source: Biomed Res Int. 2022 Sep 27;2022:8101866. doi: 10.1155/2022/8101866 (PMC9532099; doi:10.1155/2022/8101866)
Supplement: Supplementary Materials — Supplementary Table S1: list of primers used for the detection of antimicrobial resistance and virulent genes in the E. faecalis isolated from clinical mastitis. Supplementary Figure S1: phylogenetic relationship of E. faecalis isolated in this study. The relationship was inferred with the partial 16S rRNA gene sequences of E. faecalis (marked with red rectangles) obtained in this study using the Neighbor-Joining method (Saitu and Nei, 1987). The optimal tree is shown. The percentage of replicate trees in which the associated taxa clustered together in the bootstrap test (1000 replicates) is shown next to the branches (Felsenstein, 1985). The tree is drawn to scale, with branch lengths in the same units as those of the evolutionary distances used to infer the phylogenetic tree. The evolutionary distances were computed using the p-distance method (Nei and Kumar, 2000) and are in the units of the number of base differences per site. This analysis involved 15 E. faecalis and 1 E. coli sequence as an out-group. All ambiguous positions were removed for each sequence pair (pairwise deletion option). There were a total of 1452 positions in the final dataset. Evolutionary analyses were conducted in MEGA X (Kumar et al., 2018). [file 8101866.f1.docx]

**Supplementary Table S1. List of primers used for the detection of antimicrobial resistance and virulent genes in the *E. faecalis* isolated from clinical mastitis**

|  | **Target genes** | **Primer sequences (5' - 3')** |  | **Annealing temperature (^o^C)** | **Size (bp)** | **References** |
| --- | --- | --- | --- | --- | --- | --- |
| **Antimicrobials** |  | **Forward** | **Reverse** |  |  |  |
| **Aminoglycosides** | *aacC2* | GGCAATAACGGAGGCAATTCGA | CTCGATGGCGACCGAGCTTCA | 55 | 450 | Chen et al., 2004 |
|  | *aacC4* | ACTGAGCATGACCTTGCGATGCTCTA | TACCTTGCCTCTCAAACCCCGCTT | 55 | 436 | Chen et al., 2004 |
|  | *aac(6′)-Ie-aph(2′′)-Ia* | CAGGAATTTATCGAAAATGGTAGAAAAG | CACAATCGACTAAAGAGTACCAATC | 52 | 369 | Vakulenko et al., 2003 |
|  | *aph(2′′)-Ib* | CTTGGACGCTGAGATATATGAGCAC | GTTTGTAGCAATTCAGAAACACCCTT | 55 | 867 | Vakulenko et al., 2003 |
|  | *aph(2′′)-Ic* | CCACAATGATAATGACTCAGTTCCC | CCACAGCTTCCGATAGCAAGAG | 56 | 444 | Vakulenko et al., 2003 |
|  | *aph(2′′)-Id* | GTGGTTTTTACAGGAATGCCATC | CCCTCTTCATACCAATCCATATAACC | 56 | 641 | Vakulenko et al., 2003 |
|  | *aph(3")-IIIa* | GGCTAAAATGAGAATATCACCGG | CTTTAAAAAATCATACAGCTCGCG | 52 | 523 | Vakulenko et al., 2003 |
| **Tetracyclines** | *tetA* | GCGCCTTTCCTTTGGGTTCT | CCACCCGTTCCACGTTGTTA | 55 | 831 | Chen et al., 2004 |
|  | *tetB* | CCCAGTGCTGTTGTTGTCAT | CCACCACCAGCCAATAAAAT | 55 | 723 | Chen et al., 2004 |
|  | *tetC* | TTGCGGGATATCGTCCATTC | CATGCCAACCCGTTCCATGT | 55 | 1019 | Chen et al., 2004 |
|  | *tetD* | CTGGGCAGATGGTCAGATAA | TGACCAGCACACCCTGTAGT | 55 | 832 | Chen et al., 2004 |
|  | *tetE* | CGTCGCCCTGTATTGTTACT | TGGTCAGCACCCCTTGTAAT | 55 | 814 | Chen et al., 2004 |
|  | *tetG* | AGCAGGTCGCTGGACACTAT | CGCGGTGTTCCACTGAAAAC | 55 | 623 | Chen et al., 2004 |
|  | *tetK* | TTAGGTGAAGGGTTAGGTCC | GCAAACTCATTCCAGAAGCA | 50 | 718 | Aarestrup, 2000 |
|  | *tetL* | ATAAATTGTTTCGGGTCGGTAAT | AACCAGCCAACTAATGACAATGAT | 50 | 1077 | Trzcinski et al., 2000 |
|  | *tetM* | GTTAAATAGTGTTCTTGGAG | CTAAGATATGGCTCTAACAA | 50 | 657 | Aarestrup, 2000 |
| **Vancomycin** | *vanA* | ATGAATAGAATAAAAGTTGC | TCACCCCTTTAACGCTAATA | 50 | 1032 | Saha et al., 2008 |
|  | *vanB* | GTGACAAACCGGAGGCGAGGA | CCGCCATCCTCCTGCAAAAAA | 58 | 433 | Clark et al., 1993 |
| ***E. faecalis* identification** | *ddl* | ATCAAGTACAGTTAGTCTTTA | AACGATTCAAAGCTAACT | 48 | 942 | Dutka-Malen et al., 1995 |
| **Virulent genes** | *agg* | TCTTGGACACGACCCATGAT | AGAAAGAACATCACCACGAGC | 58 | 413 | Hashem et al., 2017 |
|  | *fsrA* | CGTTCCGTCTCTCATAGTTA | GCAGGATTTGAGGTTGCTAA | 53 | 474 | Hashem et al., 2017 |
|  | *fsrB* | TAATCTAGGCTTAGTTCCCAC | CTAAATGGCTCTGTCGTCTAG | 55 | 428 | Hashem et al., 2017 |
|  | *fsrC* | GTGTTTTTGATTTCGCCAGAGA | TATAACAATCCCCAACCGTG | 54 | 716 | Hashem et al., 2017 |
|  | *gelE* | GGTGAAGAAGTTACTCTGAC | GGTATTGAGTTATGAGGGGC | 52 | 704 | Hashem et al., 2017 |
|  | *sprE* | CTGAGGACAGAAGACAAGAAG | GGTTTTTCTCACCTGGATAG | 53 | 432 | Hashem et al., 2017 |
|  | *ace* | GAATGACCGAGAACGATGGC | CTTGATGTTGGCCTGCTTCC | 58 | 615 | Hashem et al., 2017 |
|  | *pil* | GAAGAAACCAAAGCACCTAC | CTACCTAAGAAAAGAAACGCG | 53 | 620 | Hashem et al., 2017 |
|  | *cyl* | TGGCGGTATTTTTACTGGAG | TGAATCGCTCCATTTCTTC | 52 | 186 | Hashem et al., 2017 |
| **Sequencing** | 16S rRNA | AGAGTTTGATCMTGGC | TACCTTGTTACGACTT | 45 | 1485 | Hahne et al., 2018 |

**References**

F. M. Aarestrup, “Occurrence, selection and spread of resistance to antimicrobial agents used for growth promotion for food animals in Denmark,” *Acta Pathologica*, *Microbiologica, et Immunologica Scandinavica. Supplementum*, vol. 108, pp. 5–6, 2000.

N. C. Clark, R. C. Cooksey, B. C. Hill, J. M. Swenson, and T. C. Tenover, Characterization of glycopeptide-resistant Enterococci from U.S. hospitals,” *Antimicrobial Agents and Chemotherapy*, vol. 37, pp. 2311–2317, 1993.

S. Chen, S. Zhao, D. G. White et al., “Characterization of Multiple-Antimicrobial-Resistant Salmonella Serovars Isolated from Retail Meats,” *Applied and Environmental Microbiology*, vol. 70, pp. 1–7, 2004.

S. Dutka-Malen, S. Evers, and P. Courvalin, “Detection of glycopeptide resistance genotypes and identification to the species level of clinically relevant enterococci by PCR,” *Journal of Clinical Microbiology*, vol. 33, pp. 24–27, 1995.

J. Hahne, T. Kloster, S. Rathmann, M. Weber, and A. Lipski, “Isolation and characterization of Corynebacterium spp. from bulk tank raw cow's milk of different dairy farms in Germany,” *PLoS ONE*, vol. 13, article e0194365, 2018.

Y. A. Hashem, K. A. Abdelrahman, and R. K. Aziz, “Phenotype–Genotype Correlations and Distribution of Key Virulence Factors in Enterococcus faecalis Isolated from Patients with Urinary Tract Infections. *Infection and Drug Resistance*, vol. 14, pp. 1713, 2021.

B. Saha, A. K. Singh, A. Ghosh, and M. Bal, “Identification and characterization of a vancomycin resistant *Staphylococcus aureus* isolated from Kolkata (South Asia)”. *Journal of Medical Microbiology*, vol. 57, pp. 72–79, 2008.

K. Trzcinski, B. S. Cooper, W. Hryniewicz, and C. G. Dowson, “Expression of resistance to tetracyclines in strains of methicillin-resistant *Staphylococcus aureus*,” *The Journal of Antimicrobial Chemotherapy*, vol. 45, pp. 763–770, 2000.

S. B. Vakulenko, S. M. Donabedian, A. M., Voskresenskiy, M. J. Zervos, S. A. Lerner, and J. W. Chow, “Multiplex PCR for detection of aminoglycoside resistance genes in enterococci”. *Antimicrobial Agents and Chemotherapy*, vol. 47, pp. 1423-1426, 2003.


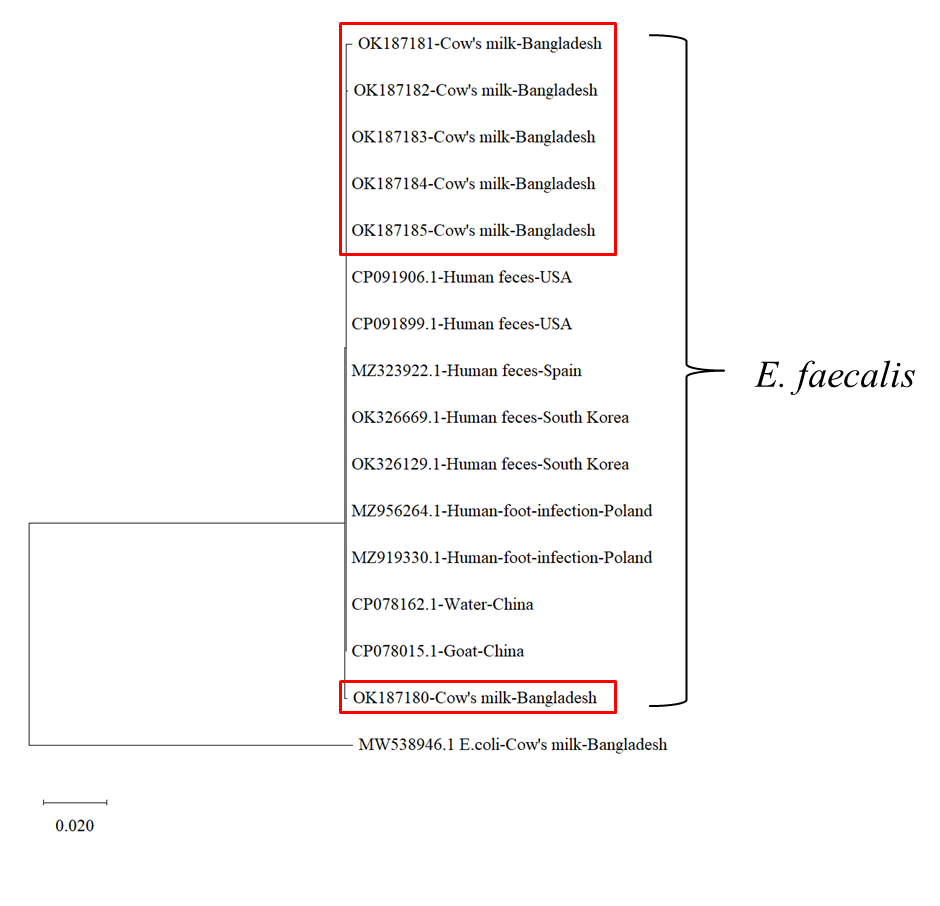


Supplementary Fig. S1. Phylogenetic relationship of *E. faecalis* isolated in this study. The relationship was inferred with the partial 16S rRNA gene sequences of *E. faecalis* (marked with red rectangles) obtained in this study using the Neighbor-Joining method (Saitu and Nei, 1987). The optimal tree is shown. The percentage of replicate trees in which the associated taxa clustered together in the bootstrap test (1000 replicates) is shown next to the branches (Felsenstein, 1985). The tree is drawn to scale, with branch lengths in the same units as those of the evolutionary distances used to infer the phylogenetic tree. The evolutionary distances were computed using the p-distance method (Nei and Kumar, 2000) and are in the units of the number of base differences per site. This analysis involved 15 *E. faecalis* and 1 *E. coli* sequence as an out-group. All ambiguous positions were removed for each sequence pair (pairwise deletion option). There were a total of 1452 positions in the final dataset. Evolutionary analyses were conducted in MEGA X (Kumar et al., 2018).

**References:**

J. Felsenstein, “Confidence limits on phylogenies: An approach using the bootstrap,” *Evolution*, vol. 39, pp. 783-791, 1985.

S; Kumar, G. Stecher, M. Li, C. Knyaz, and K. Tamura, “MEGA X: Molecular Evolutionary Genetics Analysis across computing platforms,” *Molecular Biology and Evolution*, vol. 35, pp. 1547-1549, 2018.

M. Nei, and S. Kumar, “Molecular Evolution and Phylogenetics,” Oxford University Press, New York.

N. Saitou, and M. Nei, “The neighbor-joining method: A new method for reconstructing phylogenetic trees,” *Molecular Biology and Evolution*, vol. 4, pp. 406-425, 1987.
